# Fast and low loss flexoelectro-optic liquid crystal phase modulator with a chiral nematic reflector

**DOI:** 10.1038/s41598-019-42831-5

**Published:** 2019-05-07

**Authors:** Xiuze Wang, Julian A. J. Fells, Wing C. Yip, Taimoor Ali, Jia-de Lin, Chris Welch, Georg H. Mehl, Martin J. Booth, Timothy D. Wilkinson, Stephen M. Morris, Steve J. Elston

**Affiliations:** 1 Department of Engineering Science, University of Oxford, Parks Road, Oxford, OX1 3PJ UK; 2 Department of Engineering, University of Cambridge, Cambridge, CB3 0FA UK; 3 Department of Chemistry, University of Hull, Hull, HU6 7RX UK

**Keywords:** Optoelectronic devices and components, Liquid crystals

## Abstract

In this paper, we demonstrate a flexoelectro-optic liquid crystal phase-only device that uses a chiral nematic reflector to achieve full 2π phase modulation. This configuration is found to be very tolerant to imperfections in the chiral nematic reflector provided that the flexoelectro-optic LC layer fulfils the half-wave condition. Encouragingly, the modulation in the phase, which operates at kHz frame rates, is also accompanied by low amplitude modulation. The configuration demonstrated herein is particularly promising for the development of next-generation liquid crystal on silicon spatial light modulators.

## Introduction

Electrically controllable optical phase modulation is becoming increasingly desirable in a range of applications including holography^1^, biomedical imaging^2^, aberration correction^3^, laser machining^4^ and free-space optical communications^5^. Currently, there are a number of technologies available that can be used to modulate the phase of light, this includes optical microelectromechanical systems (MEMS) and liquid crystal (LC)-based spatial light modulators (SLMs). Optical MEMS-based phase modulators containing actuators and micro-mirrors can be engineered to consume very little energy and the devices can be made very small^6,7^. These advantages make them capable of simultaneously steering a large number of optical beams. However, optical MEMs-based phase modulators typically have lower pixel densities and often lower wave-front quality control compared with rival modulator technologies^8^.

Various incarnations of LC SLMs have been developed over many years combining the merits of high-performance complementary metal oxide semiconductor (CMOS) technology with the unique electro-optical properties of LC materials. Optical phase modulation is achieved by applying an electric field across a thin LC layer which results in a corresponding change in the refractive index of the material^9,10^. Depending on the LC mesophase that is used as the active layer inside of the device, LC-SLMs can be divided into two main categories: nematic LC and ferroelectric LC. Planar aligned nematic LC-SLMs are of interest as they can provide multi-level phase modulation. However, due to the limited switching speeds available with standard nematic materials their frame rate is restricted to frequencies less than 1 kHz^10^. A fast response time is often highly sought after because it means that the device can transmit more information at relatively short timescales. Since most nematic LC devices consist of a relatively thick LC layer, which is required in order to achieve a 2π phase modulation, this results in an increase in the response time. The conventional approach to reducing the response time is to increase the electric field amplitude applied across the LC layer, but this comes at the expense of a high driving voltage, which is generally not desirable^11–15^. Ferroelectric LC SLMs, on the other hand, can offer much faster frame rates, overcoming the slower switching speeds of nematic LC technology. Unfortunately, due to the bi-stable nature of the switching mechanism in standard ferroelectric LC material device configurations, they are commonly only suitable for binary phase modulation and are typically not able to provide analogue phase modulation^16–18^.

In the ideal case, the SLM will have both multi-level (analogue) phase modulation with a fast frame rate. Towards this end, *Stockley et al*. proposed an analogue optical phase modulator based on a combination of a ferroelectric LC layer and a polymer cholesteric LC in 1995^19^. This arrangement gave an impressive 1.95π phase range at 1 kHz, but the phase was highly nonlinear with the applied voltage. More recently, a 2π phase range was reported using an anti-ferroelectric LC phase modulator, also with a fast frame rate^20^. However, the thickness of this device was 50 μm, which is not desirable from a practical perspective since thicker devices require higher driving voltages and are typically incompatible with very small pixel sizes.

**Figure 1:**
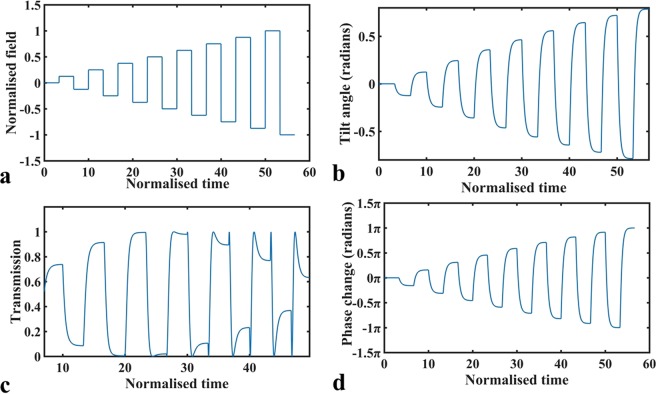
Simulation results of the flexoelectro-optic effect. (**a**) An exemplar case of an electric field applied to a ULH-aligned flexoelectro-optic device. The electric field and time are normalised to be dimensionless quantities. (**b**) The resulting tilt angle (in radians) as a function of time. (**c**) The electro-optic response of a device satisfying the half-wave condition when placed between crossed polarisers. (**d**) The resulting phase modulation for the configuration presented in this work.

Flexoelectricity in LCs was first considered by Meyer in 1969 and is a phenomenon that involves the direct coupling between the dipolar (or quadrupolar) electric polarisation and director curvature distortions^21,22^. It is commonly studied in the configuration of a Uniform Lying Helix (ULH) alignment of a chiral nematic LC whereby the helical axis of the material is aligned parallel to the device substrates^23^. When an electric field is applied to the ULH mode device (between the substrates), the coupling between the applied field and the field-induced flexoelectric polarisation introduces a distortion which causes a rotation (or tilt) of the LC director, and therefore also a macroscopic tilt of the optical axis, within the plane of the device. In our previous work^24,25^, we have investigated the electro-optic behaviour in a material that shows a high tilt angle of the optic axis when subjected to an electric field and this material (CB7CB) is used in the work presented in the current paper. The behaviour of this electro-optic effect (referred to as the flexoelectro-optic effect) is illustrated in Fig. 1, where the applied signal presented in Fig. 1(a) leads to the tilt angle response shown in Fig. 1(b). Here, dimensionless quantities of the applied electric field, *E*′, and time, *t*′, are used, whereby

1 $${E}^{\text{'}}=\{\frac{{e}_{1}-{e}_{3}}{({K}_{1}+{K}_{3})q}\}E,{\rm{and}}\,{t}^{\text{'}}=t/\tau ,{\rm{where}}\,\tau =\frac{2\gamma }{({K}_{1}+{K}_{3}){q}^{2}}$$

In this case, *K*_1_ and *K*_3_ are the splay and bend elastic constants, respectively, *e*_1_ and *e*_3_ are the splay and bend flexoelectric coefficients, respectively, and *q* = 2*π*/*p*, where *p* is the helical pitch of the chiral nematic LC that is aligned in a ULH configuration. When this structure is placed between crossed polarisers, it leads to the transmission response shown in Fig. 1(c). Here it is assumed that the layer of LC material satisfies the half-wave plate condition such that Δ*n*_*effectiove*_ d/*λ* = 0.5, where Δ*n*_*effective*_ is the optical anisotropy (birefringence) of the LC layer, *d* is its thickness and *λ* is the wavelength of light passing through the device.

LC phase modulators based on the flexoelectro-optic effect were first proposed in 2009, but in this case, they were not able to satisfy the requirement of many applications since they were only shown to provide a 0.52π phase range at 1 kHz^26^. However, recently we reported a new ULH device arrangement and experimentally demonstrated phase modulation with a full 2π phase range, together with a fast switching frame rate of 1 kHz and low residual amplitude modulation^25^. This modulator is based upon a reflective configuration which consists of a polariser, a quarter-wave plate, a ULH-aligned chiral nematic LC cell, and a reflector that preserves the handedness of circularly polarised light. In our previous work, our demonstration used a quarter-wave plate and a mirror as the reflector and was shown to provide multi-level phase modulation with fast frame rates.

In this paper, we show that multi-level phase modulation in the kHz regime can be achieved using only self-organised liquid crystalline materials without the need for a quarter-wave plate and mirror combination. Of crucial importance for the reflector component is that the axis of the helix of the chiral nematic reflector is aligned parallel to the propagation direction of light rather than perpendicular to it (as is the case for the ULH mode). This configration also has the added benefit of simplifying the device assembly making it more compatible with the silicon fabrication process and the performance is found to be tolerant to small errors in thickness of the LC device that deviates the behaviour away from that of a half-wave plate condition (provided that the chiral nematic LC reflector is ideal).

## Model of the Phase Modulator

**Figure 2:**
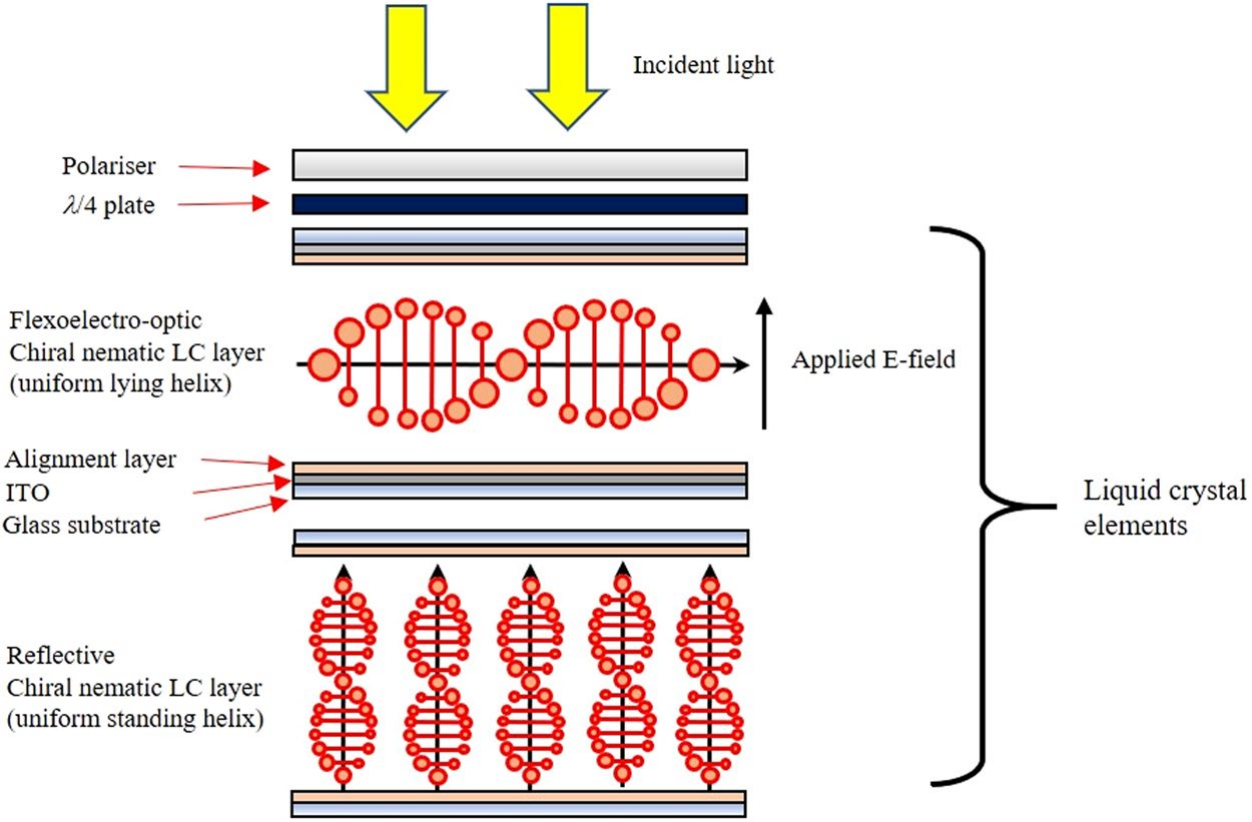
Illustration of the low-loss LC phase modulator comprising a flexoelectro-optic chiral nematic LC cell in the uniform lying helix configuration and a reflective chiral nematic LC glass cell in the uniform standing helix alignment.

The Jones Matrix method^27^ has been used in this work to model the optical behaviour of the phase modulator so as to make comparisons with the previous arrangement reported in ref.^25^. The configuration of our device reported herein (See Fig. 2) consists of a vertical polariser, followed by a quarter-wave plate, to generate circularly polarised light, which then passes through the flexoelectro-optic chiral nematic LC device, aligned in the ULH geometry and, ideally, corresponds to a half-wave plate in terms of thickness. The light is then reflected from a chiral nematic reflector before passing back though the LC device, quarter-wave plate and polariser combination. The incident polariser, quarter-wave plate and LC device can be readily modelled, whereby the LC acts as a wave-plate with an optic-axis in a plane normal to the incident beam.

The chiral nematic reflector, on the other hand, is less commonly modelled using the Jones Matrix technique. Here we model this element by combining the well-established Jones Matrices of three standard optical components – a quarter-wave plate, a linear polariser and a mirror. This is equivalent to a chiral nematic reflector because the quarter-wave plate converts the incident circular polarisation of each handedness into two linear polarisation states, the linear polariser then rejects one of these states and the transmitted linear component is subsequently reflected by the mirror. It then passes back through the linear polariser, and then through the quarter-wave plate which converts it back to the circular polarisation of one handedness only. The net effect is a component which reflects circularly polarised light of one handedness and rejects the other. Bringing all of the components together we can then write the optical output field, *E*_*out*_ as:

2 $${E}_{out}={\boldsymbol{P}}{{\boldsymbol{Q}}}_{1}(-\frac{\pi }{4}){\boldsymbol{D}}(-\phi ,\delta )\{{{\boldsymbol{Q}}}_{2}(-\frac{\pi }{4}){\boldsymbol{PMP}}{{\boldsymbol{Q}}}_{2}(\frac{\pi }{4})\}{\boldsymbol{D}}(\phi ,\delta ){{\boldsymbol{Q}}}_{1}(\frac{\pi }{4}){\boldsymbol{P}}{E}_{in}$$

Reading this equation from right-to-left, the input light *E*_*in*_ is first passed through the vertical linear polariser represented by matrix ***P***. The quarter-wave plate, the axis of which is at 45° to the vertical, is represented by a matrix ${{\boldsymbol{Q}}}_{1}(\frac{\pi }{4})$. ${\boldsymbol{D}}(\phi ,\delta )$ is then the Jones matrix of an LC device having retardance *δ* at an orientation of angle *φ* to the horizontal^28^. The term in curly-brackets then represents the chiral nematic reflector as described above, where ***M*** is the Jones matrix of a mirror and ${{\boldsymbol{Q}}}_{2}(\frac{\pi }{4})$ is a quarter-wave plate, etc. Note that in the terms after the mirror (terms to the left of *M* in Eq. (2)) the orientation angles of the wave plates are reversed because these angles are measured from the horizontal and the light is now propagating in the opposite direction. The terms to the left of the curly-brackets then represent the light passing back though the LC device, quarter-wave plate, and vertical linear polariser after it has been reflected from the reflector.

Multiplying out the terms in Eq. (2), and assuming that all components are ideal (with the LC device forming a perfect half-wave plate), and that the input and output light is vertically polarised, leads to the simple, but informative result^19^ that:

3 $${E}_{out}={E}_{in}{e}^{+4i\phi }$$

So, we can see that in this configuration pure phase modulation is obtained and that, for an angle $\phi$ that is within the range of ±π/4 (i.e. a tilt angle of the optic axis in the ULH device of ±45°, with a total switching angle range of 90°) a phase modulation range of 2π is obtained as noted previously in refs^19,25^. The resulting phase modulation for this configuration using the ULH device considered above, with an electro-optic response defined by that of Fig. 1, is shown in Fig. 1(d). It can be noted that the response has the same form as the tilt angle in Fig. 1(b), but shows a phase modulation of ±π when the tilt angle is ±45°, as expected from Eq. (3).

**Figure 3:**
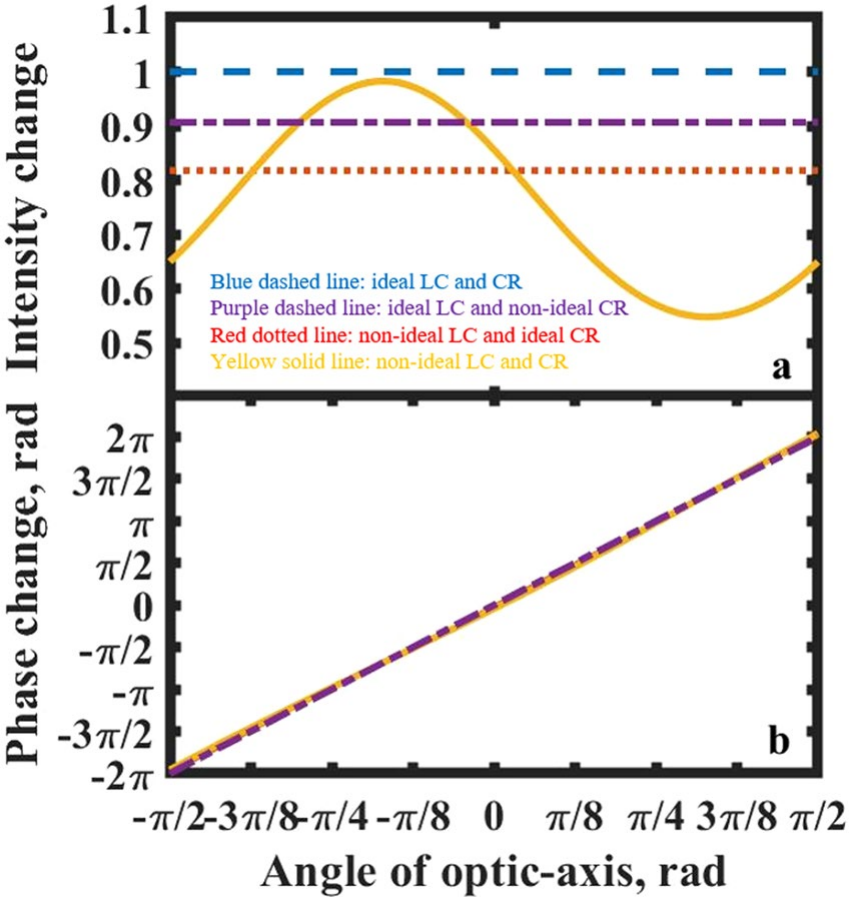
Simulations of ideal and non-ideal optical phase modulators based on the chiral nematic reflector configuration. (**a**) Intensity and (**b**) phase change are shown as a function of the absolute angle of the optic-axis of the ULH-aligned flexoelectro-optic device relative to the horizontal. The four plots shown in each case are as follows: ideal case with LC retardance of $0.5\lambda$ and ideal chiral nematic reflector (blue line dashed line); imperfect reflector and ideal LC retardance (purple dashed line); non-ideal LC retardance of $0.4\lambda$ and ideal chiral nematic reflector (red dotted line); non-ideal LC retardance of $0.4\lambda$ and imperfect reflector (yellow line).

Figure 3 shows the performance of the ideal and non-ideal optical phase modulator. The non-ideal properties of the optical phase modulator are mainly caused by two components, the LC cell and the chiral nematic reflector. In this simulation, when the LC layer is ideal, the retardance of the device is set to be 0.5λ (i.e. a half-wave plate). As explained previously, this is expected to lead to pure phase modulation of light propagating through the device. The non-ideal case is considered to be a possible error in the thickness of the ULH LC layer and is represented by setting the LC device retardance to be 0.4λ (i.e. it deviates from the half-wave condition). To represent an imperfection in the chiral nematic reflector, we “modify” the quarter-wave plate ${{\boldsymbol{Q}}}_{{\bf{2}}}$ in the terms in the curly brackets in Eq. (2). In this case, the quarter-wave plate is adjusted to have a retardance of 0.2λ (instead of the ideal quarter-wave condition (i.e. 0.25λ), and its orientation is adjusted to be at an angle of π/5 from the vertical (instead of the ideal π/4). The net result is that the reflected light from the chiral nematic “reflector” becomes slightly elliptical.

It is worth commenting on some interesting effects seen in Fig. 3. Firstly, as expected, for the ideal configuration consisting of both an ideal retardance and reflector, we have full 2π phase modulation for an optic-axis tilt angle range of π/2 of the flexoelectro-optic device (a phase modulation range of ±4π for the optic-axis tilt range of ±π/2 shown in Fig. 2b). Additionally, for the ideal case, we see that the system is lossless (the intensity is unity, indicating that all of the incident light is returned and therefore the amplitude of the reflected light is constant regardless of the angle of the optic-axis). From the figure, it can also be seen that a full 2π phase modulation range can always be realised even when one or both components is/are not ideal. Furthermore, from the intensity plots it can be seen that even if one or the other of the components are individually not perfect, the intensity change is still zero (as indicated by the red dashed and purple dashed plots in the figure). Although there is now a loss in the system, the variation in loss for both of these cases is zero, indicating that there is no modulation in the intensity. Consequently, pure phase modulation is again obtained, which is essential for many technological applications.

Substantial intensity changes (loss variation) only happens when both components are not ideal (as indicated by the yellow curve). The intensity now varies between a maximum of unity and a minimum of around 0.5 (an amplitude minimum of $\sqrt{0.5}$) for the illustrative case considered here. Hence, to minimise the intensity modulation, the required condition is that one of the components (but not both) should be “ideal”. This is an encouraging result since in practice it can be difficult to ensure that the ULH device forms an exact half-wave plate, whereas it might be possible to engineer a chiral nematic LC reflector such that the polarisation of the reflected light is close to ideal circular polarisation (by ensuring that the device is operating in the middle of the reflection band), thus providing greater flexibiltiy and tolerance in the construction of the device.

## Experimental Results

**Figure 4:**
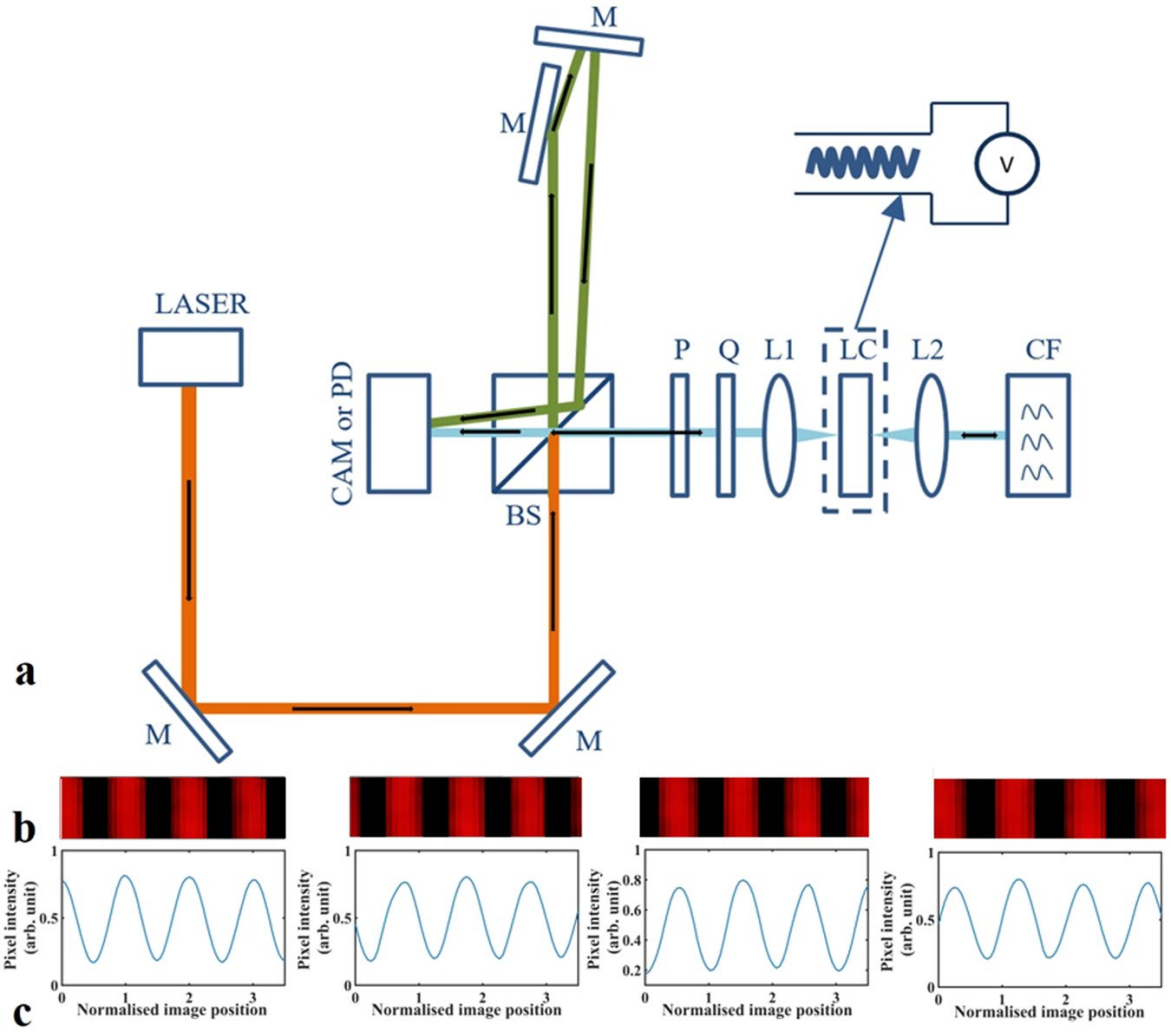
(**a**) Experimental arrangement to measure the phase and intensity response of the LC optical phase modulator. (M, mirror; BS, beam-splitter; P, polariser; Q quarter-wave plate; L1, L2, lenses; LC, modulation device; CF, chiral reflector; CAM, CCD camera; PD, photodiode.) The device is placed in one arm of a Michelson interferometer. Light is focused through the device to ensure that it passes through a mono-domain of ULH alignment. An extra mirror was introduced into the reference arm so that the light from both arms will intersect to generate clear fringes. The reflecting component in the device arm is formed from a chiral nematic LC reflector to reproduce the system outlined in the text. To measure the intensity, the CCD is replaced with a photodiode, together with a pin-hole to ensure that only the signal beam is admitted. The modulation device is a ULH flexoelectro-optic chiral nematic LC as shown. (**b**) An example set of four interference fringes recorded by the CCD camera, together with (**c**) the associated line-scans. (Note: the line-scan results shown in the figure are not single line-scans, but are averages of multiple line scans to reduce noise). These are shown for modulated phase angles at 90° intervals (the line-scans across the image are normalised to the fringe spacing).

In order to demonstrate experimentally that this configuration is indeed robust to errors in the flexoelectro-optic ULH LC layer, as the simulations suggest, a bench-top implementation of the chiral nematic reflector configuration has been assembled. For this study, we use a bimesogen-based chiral nematic LC mixture with a ULH alignment for the modulating element (via the flexoelectro-optic effect) and a second chiral nematic LC in a standing helix (Grandjean) alignment for the reflector. The optical phase shift and intensity response were measured using a Michelson interferometer, as illustrated in Fig. 4.

The implementation of the chiral nematic reflector-based phase modulator is also shown in Fig. 4a. This implementation consists of the components explained previously, as represented in Eq. (2). First, there is a vertical polariser (P), followed by a quarter-wave plate (Q). Together, the combination generates circularly polarised light, which was confirmed using a polarisation analyser (Schäfter + Kirchhoff SK010PA-VIS). The light then passes through the modulating device which is a chiral nematic LC in the ULH state that undergoes flexoelectro-optic switching (this was the term ${\rm{D}}({\rm{\phi }})$ in Eq. (2)). The device was placed on a temperature controlled hot-stage to allow it to be operated in the chiral nematic phase, which occurs at an elevated temperature (>100 °C). The light is then reflected from the chiral nematic reflector (CF) (the term in curly brackets in Eq. (2)) and subsequently passes back through the same system. A pair of lenses (L1 and L2) were included to ensure that the light only passes through a mono-domain region of the LC device.

Taking the ordinary and extraordinary refractive indices for the nematic LC, CBC7CB, at 106 °C and a wavelength of 633 nm from previously published work^29^, and using a standard expression for the effective optical anisotropy of a ULH structure^30^, the retardance for the ULH device used here was estimated to be 0.51λ. Since the retardance of the LC device is approximately 0.5λ, we consider it to behave as a near-ideal half-wave plate, which reverses the hand of circular polarised light passing through it. The mixture for the chiral nematic reflector was composed of the nematic LC, HTW114200-100, (Fusol) and 22.2 wt% of the left-handed chiral dopant, S811 (Merck). This mixture was filled into a cell and was found to assume a Grandjean texture (whereby the helix axis is aligned parallel to the normal of the glass substrates) on a polarising optical microscope. Measurements on a UV-Vis spectrometer confirmed that the centre of the reflection band was close to the wavelength of the He-Ne laser (632.8 nm). Figure 4b shows example images of the interference fringes recorded by the CCD camera. Results are shown for four different applied voltages, representing four different phase modulation states. It should be noted that multiple line-scans are taken across the fringes in the images and then averaged to reduce noise and provide useful line-scan data. It can be seen from the line scans in Fig. 4(c) that as the optical phase changes the fringes move. To extract the modulated optical phase shift from this data a simple fitting procedure was used^25^.

**Figure 5:**
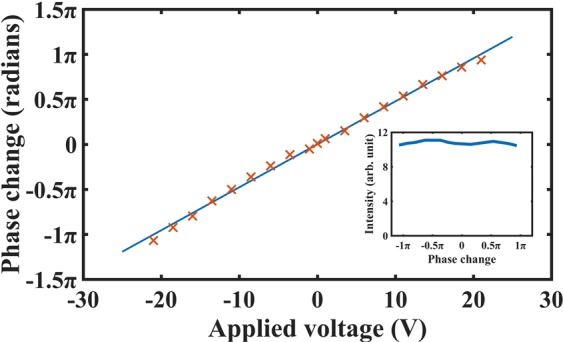
Experimentally determined phase and intensity for the LC optical phase modulator presented in this work (measured using the system presented in Fig. 4 with a CCD camera and photodiode). The main plot shows the optical phase shift as a function of voltage. The lower right inset shows the intensity variation as a function of the measured optical phase angle. The variation in intensity is seen to be minimal.

Figure 5 shows the experimental results for the optical phase shift and intensity variation of the new configuration. The applied voltage was varied up to ±21 V, at which point the phase range was found to be almost 2π (actually measured to be 361.4°). The phase change as a function of voltage follows an almost linear behaviour, as may be expected from the model results shown in Fig. 3. The slight nonlinearities seen in the optical phase behaviour have two origins: (i) the flexoelectro-optic tilt angle in the ULH device is not precisely linear with voltage; (ii) small drifts in environmental temperature and corresponding air currents (caused principally by the hot-stage stabilising the device temperature) lead to small drift errors in the fringe phase. It can also be seen in the inset shown in Fig. 5 that there is very little modulation in the intensity (and hence the amplitude) of the light. So, as anticipated this device configuration with a chiral nematic reflector is operating well as a pure phase modulation system. The small changes in intensity that are observed are likely to be due to the electric field-dependence of the birefringence of the ULH device.

In terms of efficiency, it was found that this varied between 16% and 44%, depending upon the alignment quality. The relatively low efficiency is due to scattering from non-uniformities in the lying helix alignment of the LC phase modulation layer and it is expected that the maximum efficiency could be significantly improved through the use of high quality ULH alignments such as those formed using solvent-assisted processes^31^. It is also worth stating that for a practical device with a silicon backplane, any unmodulated light would be reflected by the silicon backplane, pass through the LC components and would then be blocked by the polariser at the front of the device. While this would affect the overall transmission efficiency, it does not affect the phase modulation properties.

## Conclusions

In summary, we have demonstrated an LC optical phase modulator based on the flexoelectro-optic behaviour of a ULH-aligned chiral nematic LC combined with a chiral nematic reflector. This configuration exhibits full 2π phase modulation and minimal intensity variation by applying a 4 V/μm electric field at a temperature of 106 °C. In the future, this configuration can be integrated into a compact device design with a reflecting back-plane that consists of a chiral nematic LC. This design has substantial potential in spatial light modulator technology, enabling full 2π phase modulation with low-intensity modulation. Furthermore, through this research, it has been shown that an ideal chiral nematic reflector ensures that the system is less sensitive to the non-ideality in the LC phase modulation element. This means that the chiral nematic reflector needs to be carefully designed in order to reduce the amplitude modulation in the system. While a 4 V/μm electric field and an operating temperature of 106 °C are still not yet suitable for commercial SLM devices, these limitations can be addressed through the development and refinement of the liquid crystalline materials and mixtures to obtain larger *e/K* ratios and larger *∆n*.

## Methods

### Phase modulation measurements

The light source used in this setup was a continuous wave Helium-Neon (He-Ne) laser (Uniphase 1125P) that generates light at a wavelength of 632.8 nm. As illustrated in Fig. 4a), the input light is arranged to be vertically polarised and then passes via the aid of mirrors through a non-polarising beam-splitter (Newport 05BC16NP). One of the output beams (the signal beam) passes through the ULH flexoelectro-optic device and is reflected by the chiral nematic reflector. The other output beam (the reference beam) is directed by two mirrors to reflect the light back with a small angle offset so as to generate clear interference fringes. The fringes were captured by using a collection lens to image directly onto a CCD camera (Thorlabs DCU224C, 1280 × 1024, 8-bit colour).

An arbitrary function generator (Wavetek 395) was used to drive the ULH flexoelectro-optic device through an additional voltage amplifier (FLC Electronics F10AD) so as to meet the required drive voltage. The function generator also triggered the acquisition of images from the CCD camera. For all measurements, the device drive signal was a 1 kHz square-wave with a controllable amplitude level, and the camera was triggered on one half-cycle of the square-wave in order to record the instantaneous fringe intensity pattern at any desired drive voltage level. The CCD camera was set to record a 100 μs exposure towards the end of a half-cycle of the square-wave.

### Sample fabrication

The LC mixture used in the flexoelectro-optic device contained the bimesogen (dimer) CBC7CB [4′,4′-(heptane-1,7-diyl)bis(([1′,1″-biphenyl]-4″-carbo-nitrile))] dispersed with 3 wt% of the high twisting power chiral dopant, R5011 (Merck Ltd). The mixture was then filled into a nominally 5 μm-thick Instec cell that contained antiparallel rubbed polyimide alignment layers and indium tin oxide electrodes. The thickness of the empty cell was determined to be 4.73 μm by measuring the white-light transmission spectrum on a UV-Vis spectrometer (Agilent 8454). The LC mixture was found to exhibit a right-handed, chiral nematic phase between 102 °C and 118 °C (on heating). To obtain a ULH configuration, the cell was first heated to 120 °C (above the clearing temperature) and then cooled down in the presence of a 1 kHz, ±20 V square-wave signal (applied to the cell electrodes). All subsequent measurements were carried out with the device in the chiral nematic phase at a temperature of 106 °C. At this temperature, the switching time (10–90% response time) of the device was found to be approximately 120 μs.
